# Post-transcriptional regulator Rbm47 elevates IL-10 production and promotes the immunosuppression of B cells

**DOI:** 10.1038/s41423-018-0041-z

**Published:** 2018-05-29

**Authors:** Yinxiang Wei, Fanghui Zhang, Yu Zhang, Xiaoqian Wang, Chen Xing, Jing Guo, Hui Zhang, Zhimin Suo, Yan Li, Jianli Wang, Renxi Wang, Zhijian Cai

**Affiliations:** 1 Institute of Immunology, Zhejiang University School of Medicine, Hangzhou, 310058 China; 2 Laboratory of Immunology, Institute of Basic Medical Sciences, Beijing, 100850 China; 3 Joint National Laboratory for Antibody Drug Engineering, Institute of Immunology, Henan University School of Medicine, Kaifeng, 475004 China; 4 Department of Gastroenterology, Huaihe Hospital of Henan University, Kaifeng, 475000 China; 5 Translational Research Institute, Henan Provincial People‘s Hospital, Zhengzhou, 450003 China

## Abstract

Regulatory B cells (Bregs) are a functionally defined B cell subset, and IL-10 is crucial for the suppressive functions of Bregs. However, little is known regarding how IL-10 production is regulated in B cells. To explore the mechanisms by which IL-10 is regulated in B cells, we used mRNA microarrays to screen for molecules that are upregulated in IL-10-producing B cells and identified RNA-binding motif protein 47 (*Rbm47*) as a post-transcriptional regulator. Rbm47 was found to promote IL-10 production in B cells. We found that Rbm47 promotes the stability of IL-10 mRNA by binding to AU-rich elements in the 3′ untranslated region of *Il10* mRNA. In addition, we demonstrated that the overexpression of Rbm47 enabled B cells to facilitate Foxp3^+^ regulator T-cell induction and reduce the severity of DSS-induced ulcerative colitis. Taken together, these results suggest that Rbm47 plays an important role in regulating IL-10 at the post-transcriptional level, thus promoting the regulatory functions of B cells. The findings presented in this study not only increase our understanding of the post-translational regulation of IL-10 in B cells but also identify a novel strategy for the potential application of Bregs.

## Introduction

It has long been accepted that B cells have a central role in immune-related diseases because of their ability to produce antibodies.^1–4^ However, in recent decades, investigators have discovered that B cells have unique immune-suppressive abilities; since then, a substantial amount of work has been conducted to elucidate the characteristics of Bregs, a functional B-cell subset that has been proposed to negatively regulate immune responses and maintain immune homeostasis.^3, 5, 6^

Although Bregs have attracted the interest of many immunologists and clinicians following the advent of B-cell depletion therapy,^2^ the lack of lineage-specific markers makes it difficult to understand the characteristics and development of Bregs, thus hampering the practical application of this B-cell subset.^2, 7, 8^ It has been demonstrated that immature B cells, mature B cells, and plasma cells have the capacity to differentiate into IL-10-producing Bregs in both mice and humans, suggesting that Bregs are not derived from specific precursors but arise from B cells at unrestricted development stages.^9^

Despite numerous reported mechanisms, Bregs largely exert their regulatory function through IL-10, one of the most relevant cytokines involved in anti-inflammatory responses.^10–12^ IL-10 produced by Bregs participates in various regulatory processes, including the maintenance of regulatory T-cell (Treg) differentiation and the inhibition of pro-inflammatory cytokine release, pathogenic T-cell response, and antigen presentation.^13^ IL-10-producing Bregs can exert therapeutic effects in many disease models, while IL-10-deficient Bregs cannot.^6, 14–16^

It will be of great scientific and clinical significance if detailed knowledge can be obtained regarding how IL-10 production is regulated in B cells. NF-κB and Blimp1 have been found to regulate IL-10 transcription in B cells.^17^ However, it is unclear which protein regulates IL-10 at the post-transcriptional level. To explore this mechanism in B cells, we screened for key molecules that differ between IL-10-producing B cells and non-producing B cells using mRNA microarrays. We identified Rbm47, an RNA-binding protein with a role in the immune system that is not clearly understood, as an unregulated transcriptional regulator that is specific to IL-10-producing B cells. Further study indicated that Rbm47 could directly bind AU-rich elements (AREs) in the 3′-untranslated region (3′UTR) and delay the degradation of *Il10* mRNA. Importantly, Rbm47 overexpression enabled B cells to induce Tregs and decrease inflammation in mice suffering from intestinal injury. Taken together, these findings suggest that Rbm47 can positively regulate IL-10 production at the post-transcriptional level and upregulate the immune-suppressive function of B cells.

## Materials and methods

### Mice and reagents

C57BL/6 mice were purchased from The Chinese Academy of Medical Sciences (Beijing, China). The IL-10-EGFP reporter Tiger mice^18^ were kindly provided by Prof. Lie Wang (Institute of Immunology, Zhejiang University School of Medicine, Hangzhou, China). All mice were bred in our animal facility under specific pathogen-free conditions.

LPS (*Escherichia coli* 055:B5), actinomycin D (cat. no. A4262), puromycin (cat. no. 58-58-2), PMA (cat. no. P-8139), and ionomycin (cat. no. I-0634) were obtained from Sigma (St. Louis, MO, USA). Antibodies against β-Actin, GFP, and IgG were form Santa Cruz Biotechnology (Santa Cruz, CA, USA), and antibodies against Rbm47 were obtained from Abcam (Cambridge, UK). Fluorescently labeled antibodies against IL-10 (PE), B220 (APC), CD4 (FITC), and Foxp3 (PerCP) were obtained from eBioscience (San Diego, CA, USA), and antibodies against α4β7 (APC) were obtained from Biolegend (San Diego, CA, USA).

### Agilent gene array scanner (Affymetrix) microarrays

Bregs and IL-10-negative B cells were sorted from LPS-stimulated B cells using Breg-specific microbeads (cat. no. 130-095-873, AutoMACS, Miltenyi Biotec). Total RNA was extracted from B cells with TRIzol and purified using Qiagen RNeasy columns (Qiagen). Synthesis and labeling of RNA and hybridization of arrays were performed. Labeled arrays (Qiming Corp., Shanghai, China) were scanned on an Affymetrix.

### Plasmids

The mouse *Rbm47* (GenBank Accession number NM_178446) sequence was derived via PCR-based amplification from LPS-stimulated B cells and subcloned into the retroviral vector pMX-IRES-GFP or pcDNA3.1 using EcoRI and NotI. The primers used were as follows: sense, 5′-GAATTCATGACTGCTGAAGATTCCGCC-3′ and antisense, 5′-GCGGCCGCTCAGTAAGTCTGGTAGACGTC-3′. The mouse *Il10* (GenBank Accession number NM_010548) full-length cDNA-containing pcDNA3.1 vector was obtained from Genomics Technology Ltd. (Guangzhou Province, China). The Rbm47-GFP fusion plasmid pEZ-Rbm47-Lv122 and control-GFP vector pEZ-Lv122 carrying a CMV promoter were obtained from Funeng Ltd. (Guangzhou Province, China). The luciferase reporter vector pGL3 containing *Il10* promoter sequence was generated in our laboratory.

### Retrovirus packaging

pMX-IRES-GFP and pMX-*Rbm47-IRES-GFP* plasmids were transfected into Platinum-E cells via the classical calcium phosphate coprecipitation technique to generate empty vector-containing or Rbm47-expressing retrovirus according to a previously described protocol.^19^

### Overexpression of Rbm47 in B cells

The overexpression of Rbm47 in primary B cells from Tiger mice was achieved via lentiviral or retroviral infection. For lentiviral infection, B220^+^ B cells (1 × 10^6^ cells/ml) were cultured for 1 day in 6-well plates (total volume: 5 ml) in RPMI 1640 (HyClone, UT, USA) medium containing 10% FBS in the presence of 1 ml empty vector-containing or Rbm47-expressing lentivirus (Funeng Ltd., Guangzhou Province, China). The infected cells were washed and cultured for another 3 days in 6-well plates (at 1 × 10^6^ cells/ml, total volume: 5 ml) in the presence of 10 μg/ml LPS. On day 3, the cells were collected for further experiments. For retroviral infection, B220^+^ B cells were pre-stimulated with 10 µg/ml LPS for 12 h in 24-well plates (1 × 10^6^ cells/ml, total volume: 2 ml). The culture medium was then removed, and 1 ml empty vector-containing or *Rbm47*-expressing retrovirus was added to each well together with 5 µl liposomes. The mixtures were centrifuged at 2000 rpm for 75 min at 32 °C. The infected cells were washed and cultured for another 3 days in 24-well plates (1 × 10^6^ cells/ml, total volume: 2 ml) in the presence of 10 μg/ml LPS. On day 3, the cells were collected for further experiments.

### RNA immunoprecipitation

HEK-293T cells were transfected with *Rbm47*-*GFP* fusion plasmid and control-GFP plasmid. After 2 days, the cells were harvested and subjected to RNA-binding protein immunoprecipitation (RIP) with monoclonal antibody against GFP or IgG according to the manufacturer’s instructions (cat. no. 17-701, Millipore). The precipitated mRNA was then reverse transcribed into cDNA and subjected to quantitative PCR (q-PCR) analysis. The data were expressed as fold enrichment of anti-GFP Ab relative to IgG.

### Luciferase reporter assay

The luciferase reporter vectors containing full-length *Il10*, 5′UTR, *Il10*, CDS, and *Il10* 3′UTR were generated by amplifying the corresponding fragments and cloning them into the PGL3-promoter vector at KpnI and XhoI restriction sites (Promega, Madison, WI). HEK-293T cells were co-transfected with 100 ng luciferase reporter plasmids, 10 ng thymidine kinase promoter-Renilla luciferase reporter plasmid, and the *Rbm47*-pcDNA3.1 or control vector. After 48 h, luciferase activity was determined using the Dual-Luciferase Reporter Assay System (cat. no. E10910, Promega) according to the manufacturer’s instructions. The primers were as follows: *Il10* 3′UTR sense, 5′-GGGTACCGGTACCAACACCTGCAGTGTGTATTGAGT-3′, antisense, 5′-CCGCTCGAGCGGCCGCCGAATAAGA-3′; *Il10* 5′UTR sense, 5′-GGGTACCACATTTAGAGACTTGCTCTTGCACTACCAAAGCCACAAGGCAGCCTTGCAGAAAAGAGAGCTCCATCCTCGAGCGG-3′, antisense, 5′-CCGCTCGAGGATGGAGCTCTCTTTTCTGCAAGGCTGCCTTGTGGCTTTGGTAGTGCAAGAGCAAGTCTCTAAATGTGGTACCC-3′; *Il10* CDS sense, 5′-GGGTACCATGCCTGGCTCAGCACTG-3′, antisense 5′-CCGCTCGAGTTAGCTTTTCATTTTGATCATCAT3′; *Il10* 3′UTR-dARE sense, 5′-ACACCATTCCCAGAGGAATTGTACAAACGAGGTTTTCCAA-3′, antisense, 5′-TTGGAAAACCTCGTTTGTACAATTCCTCTGGGAATGGTGT-3′.

### Preparation of Rbm47-knockdown B cells

The B-cell line SP2/0 was cultured for 1 day in 24-well plates (1 × 10^5^ cells/ml, total volume 2 ml) in RPMI 1640 medium containing 10% FBS, 2 mM glutamine, penicillin (100 IU/ml), streptomycin (100 mg/ml), and 50 mM 2-ME in the presence of 100 µl control lentivirus or sh-*Rbm47* lentivirus (Santa Cruz, CA, USA). The infected cells were washed and cultured for another 7 days in 24-well plates (1 × 10^5^ cells/ml, total volume: 2 ml) in the presence of 5 μg/ml puromycin. Then, resistant SP2/0 cell clones with low levels of Rbm47 were selected by limiting dilution, confirmed and characterized by q-PCR and western blotting.

### *Il10* mRNA decay assay

To analyze the *Il10* mRNA decay rate, the Rbm47-knockdown SP2/0 cells mentioned above and control SP2/0 cells were stimulated with 10 µg/ml LPS for 6 h. Actinomycin D (10 mg/ml) was added, and total RNA was harvested after 0, 15, 30, 45, or 60 min. *Il10* mRNA levels were measured using q-PCR and normalized against the level of *gadph* mRNA. The normalized *Il10* mRNA level at time point 0 was set to 100.

### Cell sorting

IL-10-positive or IL-10-negative B220^+^ cells derived from Tiger mice and *GFP*-expressing and *Rbm47-GFP*-expressing retrovirus-infected B cells derived from C57BL/6 mice were acquired using the FACSAria system (BD Biosciences) depending on GFP expression and gating based on live lymphocyte-sized cells.

### Western blot analysis

A total of 20 µg total protein extracted from cell lysates was separated using SDS-PAGE and transferred onto polyvinylidene difluoride membranes (Millipore, Billerica, MA). The blots were probed with corresponding primary Abs, and HRP-conjugated secondary F(ab′)2 antibodies (Zymed Laboratories, San Francisco, CA) were used; the signals were visualized with the ECL detection system (Amersham, Arlington Heights, IL). The membranes were finally scanned using a Tanon 4500 (Shanghai, China) according to the manufacturer’s instructions.

### RNA isolation and q-PCR

Total RNA was extracted using TRIzol reagent (cat. no. 15596026, Invitrogen). Reverse transcription (cat. no. DRR063A, Takara) and q-PCR (cat. no. DRR041A, Takara) were performed according to the manufacturer’s instructions. Q-PCR was performed on an iQ5 PCR system (Bio-Rad, CA, USA), and gapdh was used as the internal reference. The primers used are as follows: Mus *Il10*, sense, 5′-TACACCTGCGTTTCTCAGCC-3′, antisense, 5′-CAGTATTGCACTCTGTAAGCCC-3′; Mus *Rbm47* sense, 5′-AGCCATGAACAGCGATCCAAC-3′, Antisense, 5′-CCGGTGCGCTCTATCAGTG-3′; Mus *gapdh* sense, 5′-CTGAGTATGTCGTGGAGTCT-3′, antisense, 5′-GTGGATGCAGGGATGATGTT-3′

### Flow cytometric analysis and intracellular cytokine staining

Cells (1 × 10^6^ cells/sample) were washed with FACS staining buffer (PBS, 2% FBS or 1% BSA and 0.1% sodium azide). All samples were incubated with anti-FcR antibody (clone 2.4G2; BD Biosciences, San Jose, CA, USA) before incubation with other antibodies diluted in FACS buffer and supplemented with 2% anti-FcR antibody. For intracellular cytokine staining, 50 ng/ml PMA and 1 mg/ml ionomycin were added, followed by 1 mg/ml brefeldin A and 2 mM monensin 3 h later. After 3 h, the cells were collected and fixed for 20 min with 1 ml fixation buffer (Intracellular Fixation & Permeabilization Buffer Kit; eBioscience). After washing, the fixed cells were stained with the corresponding fluorescently labeled antibodies. Data collection and analyses were performed on a FACSCalibur flow cytometer using CellQuest software (BD Biosciences).

### Cytokine analysis by ELISA

The concentration of IL-10 in cell culture supernatants was measured using ELISA kits (cat. no. 88-710522, eBioscience).

### In vitro B–T-cell coculture

Normal mouse spleen-derived CD4^+^ T cells, at a concentration of 4 × 10^5^ cells/ml, were cocultured in 96-well plates with 4 × 10^5^ cells/ml FACS-sorted B cells infected with GFP-expressing or *Rbm47*-*GFP*-expressing retrovirus in the presence of CD3 and CD28 stimulation. After 72 h, the cells and culture supernatant were collected and analyzed using FACS and ELISA, respectively.

### Induction and treatment of DSS-induced intestinal injury

Six-week-old male C57BL/6J mice were used for the induction of experimental colitis. The mice were randomly distributed into four groups (*n* = 5) and acclimatized for 1 week in our animal facility before DSS administration. The induction and evaluation of DSS-induced intestinal injury was modified from previously described methods.^17^ For colitis induction, normal drinking water was replaced with sterilized reverse osmotic water containing 3% (w/v) dextran sulfate sodium (DSS, molecular mass 36–50 kDa, MP Biomedicals, OH, USA). For treatment, GFP-expressing or *Rbm47*-*GFP*-expressing retrovirally infected B cells, termed B-Ctrl and B-Rbm47, respectively, were sorted using FACS and injected into mice via tail veil injection with PBS (2 × 10^5^ cells/mouse, 5 mice/group) on days 1 and 3 after DSS feeding. The water consumption per mouse was monitored daily and was comparable among the groups. DSS-induced intestinal injury was mainly determined based on weight loss. On day 7 after DSS induction, the mice were euthanized to assess colonic damage. The colons were removed from the mice, and colon length was measured as a marker of intestinal injury. The colons were cleaned, and the stool was flushed from each sample with ice-cold PBS. The colon samples were segmented and fixed in 10% buffered formalin for histological analyses. After paraffin embedding, 5-µm-thick cross-sections were cut and stained with H & E and observed microscopically. The degree of intestinal injury was determined based on leukocyte infiltration, crypt damage, wall thickening, and loss of goblet cells.

### Statistical analysis

Statistical significance was determined by the unpaired *t*-test for two groups and one-way ANOVA for multiple groups with GraphPad Prism (version 5.0; GraphPad Software), and values are represented as the mean ± SEM. The results were considered statistically significant at *P* < 0.05.

## Results

### Rbm47 expression in IL-10-producing B cells

**Fig. 1:**
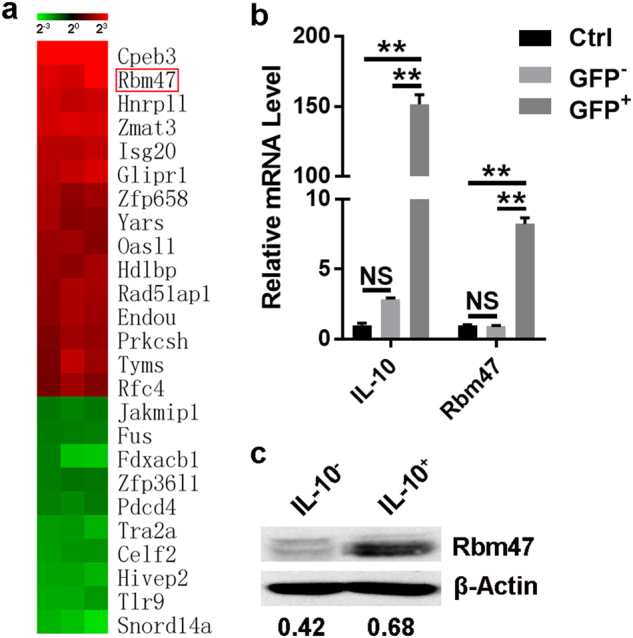
Rbm47 expression is increased in IL-10-secreting B cells. **a** IL-10^+^ and IL-10^−^ B cells from LPS-stimulated B cells were isolated by MACS, and the mRNA expression profiles were analyzed via Affymetrix microarrays. The heat map diagram shows the fold change in the expression of different genes related to RNA-binding (only differentially expressed mRNAs with a fold change ≥2.50 or ≤0.40 and *P* value < 0.05 are included). The red box indicates *Rbm47*. **b**, **c** B cells from splenocytes of *Il10*-GFP reporter mice were stimulated with or without (Ctrl) 10 μg/ml LPS. On day 3 after stimulation, GFP^−^ and GFP^+^ B cells were sorted via FACS and then subjected to q-PCR (**b**) or western blot (**c**) analysis. Numbers indicate the ratio of gray values of the corresponding protein to that of β-actin. Data are representative of at least three independent experiments, and error bars indicate the standard deviation. **P < 0.01; NS not significant

To explore the specific molecules that might regulate IL-10 expression in B cells, we isolated IL-10^+^ and IL-10^−^ B cells (with purity of ~95%) from LPS-stimulated B cells with a Breg isolation kit (AutoMACS, Miltenyi Biotec) and analyzed the cells via Affymetrix microarrays. Compared with IL-10^−^ B cells, there were 715 upregulated genes and 599 downregulated genes in IL-10^+^ cells (Supplementary Table 1). Among the different expression genes related to RNA binding, *Rbm47* was one of the most upregulated genes in IL-10^+^ B cells along with IL-10 (Fig. 1a). We detected Rbm47 expression in B cells stimulated with LPS, a common method to induce IL-10 production in B cells. The results showed that Rbm47 expression was significantly increased at both protein and mRNA levels during the induction of IL-10 production in B cells (Supplementary Figure 1a and b). However, because the separation of IL-10^+^ or IL-10^−^ B cells with microbeads depends on the binding of IL-10 on the surface of B cells, the sorting efficiency was limited. IL-10 reporter Tiger mice have been developed to examine IL-10 expression. In Tiger mice, an internal ribosomal entry site-GFP construct follows the genomic *Il-10* coding sequence, resulting in cytoplasmic expression of GFP during IL-10 induction.^18^ Moreover, no gene dose-dependent decrease in IL-10 production was observed in the B cells of homozygous Tiger mice,^20^ a phenomenon that is observed in T cells. We stimulated B cells isolated from the spleen of Tiger mice followed by FACS, and IL-10^+^ and IL-10^−^ B cells were separated based on GFP expression (with a purity of ~99%) (Supplementary Figure 1c). The cells were then subjected to q-PCR and western blotting analysis. The results showed that Rbm47 expression was higher in the IL-10-producing B cells (Fig. 1b,c), which further confirmed our above findings.

### Knockdown of Rbm47 impaired IL-10 production in B cells

**Fig. 2:**
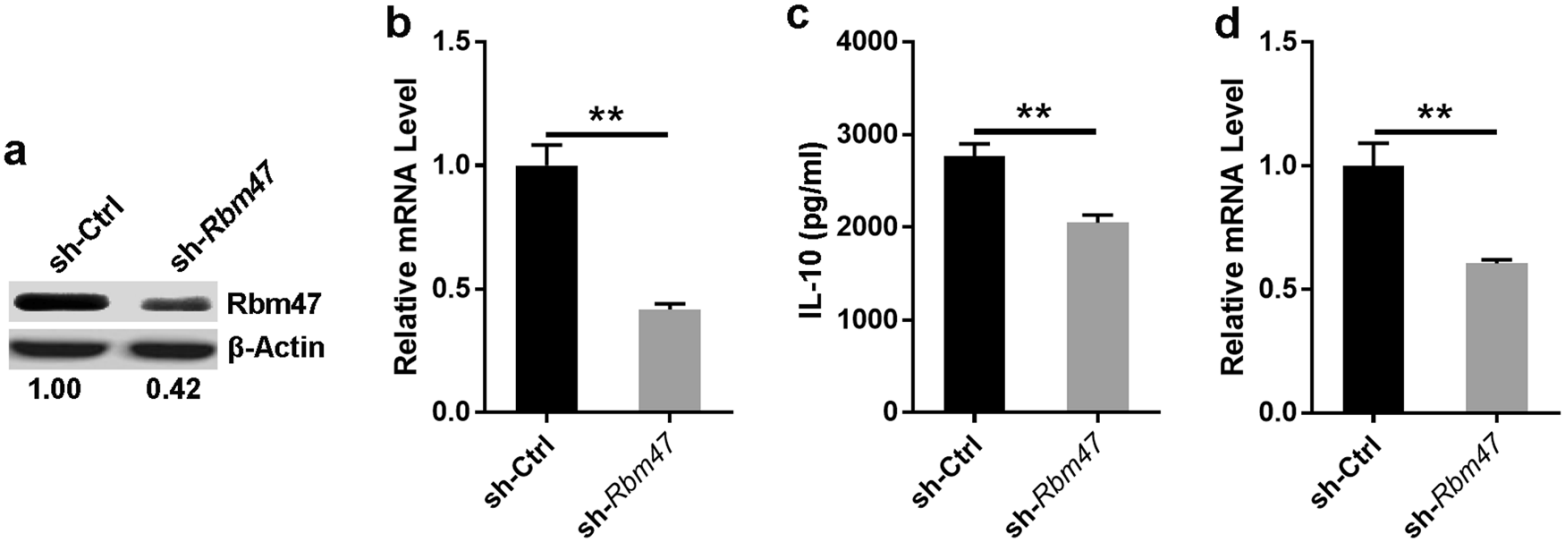
Knockdown of Rbm47 impairs IL-10 production. **a**, **b** SP2/0 cells were infected with sh-*Rbm47*-expressing or control lentivirus, and the knockdown efficiency was assessed by western blotting (**a**) and q-PCR (**b**). **c**, **d** Cells from different groups were cultured for 3 days in the presence of 10 μg/ml LPS, and then the level of IL-10 was detected using ELISA (**c**) and q-PCR (**d**). Numbers indicate the ratio of gray values of the corresponding protein to that of β-actin. Data are representative of at least three independent experiments, and error bars indicate the standard deviation. **P < 0.01

To explore the influence of Rbm47 on IL-10 production in B cells, we attempted to knock down Rbm47 expression in B cells through *Rbm47*-specific shRNA-expressing lentiviral infection. We performed the experiment using the SP2/0 cell line, which was shown to be a plasma B cell line with the ability to simultaneously secrete IL-10 and express Rbm47 in our preliminary experiments (Supplementary Figure 2a, b). We infected SP2/0 cells with *Rbm47*-specific shRNA-expressing lentivirus followed by puromycin selection. Western blotting and q-PCR analysis demonstrated that Rbm47 expression was dramatically decreased in SP2/0 cells infected with *Rbm47*-specific shRNA-carrying lentivirus (Fig. 2a, b). We did not observe any differences in cell survival between control shRNA and *Rbm47*-specific shRNA-infected SP2/0 cells (Supplementary Figure 3). As expected, the ELISA results also showed that the level of IL-10 protein was decreased in the Rbm47-knockdown SP2/0 cells (Fig. 2c). Furthermore, q-PCR analysis revealed that *Il10* mRNA was significantly reduced with Rbm47 knockdown (Fig. 2d). Collectively these results indicated that Rbm47 depletion downregulated IL-10 mRNA and protein in B cells.

### Interaction between Rbm47 and *Il10* mRNA

**Fig. 3:**
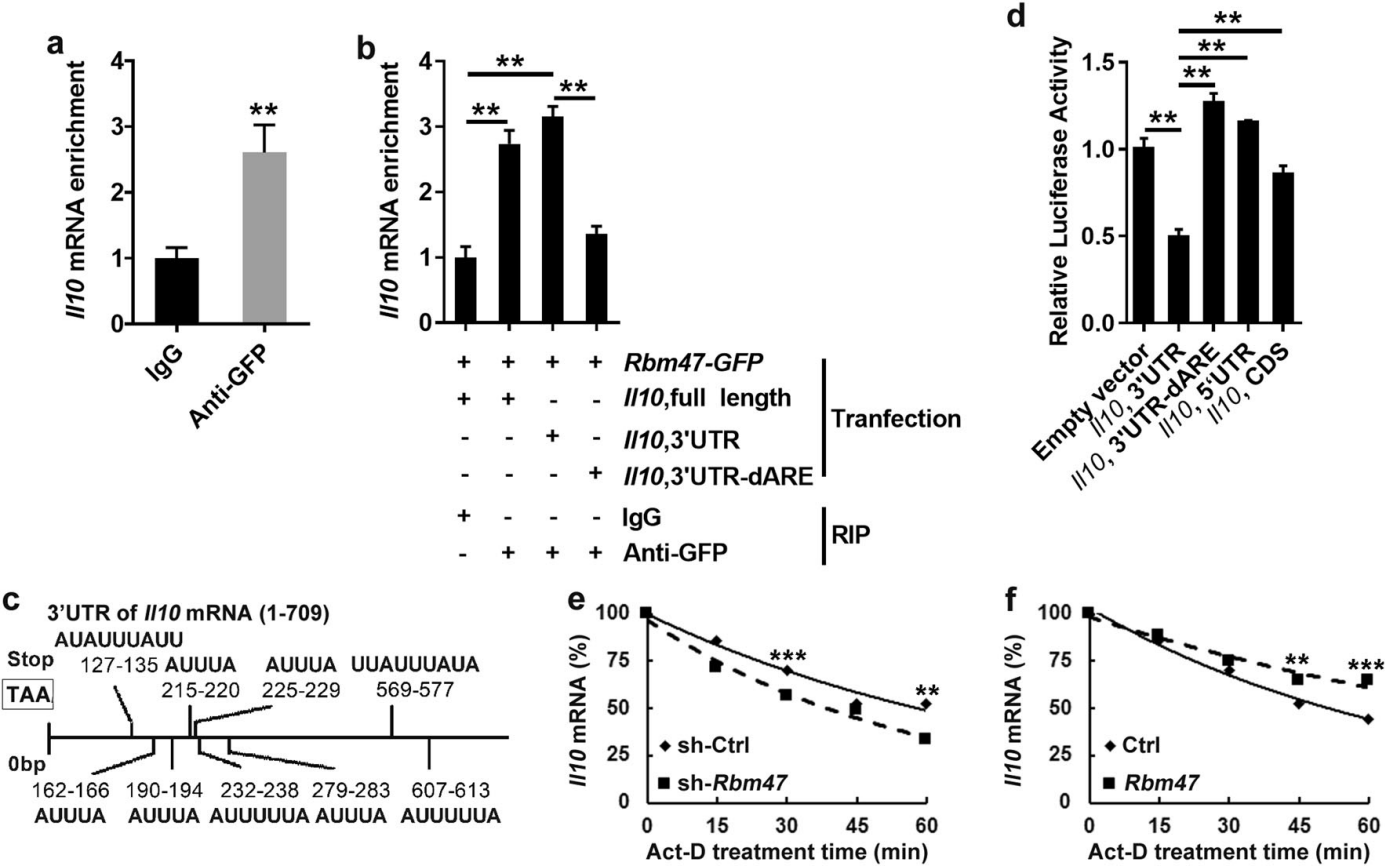
Interaction between Rbm47 and Il10 mRNA. **a**, **b** HEK-293T cells were transfected with GFP- or *Rbm47*-*GFP*-expressing plasmid (**a**). HEK-293T cells were transfected with plasmids containing *Rbm47-GFP* with *Il10*, full-length, *Il10*, 3′UTR or *Il10*, 3′UTR-dARE (**b**). After 2 days, the cells were harvested and subjected to RIP with IgG and anti-GFP antibody. The precipitated mRNA was then reverse transcribed into cDNA and subjected to q-PCR analysis. **c** AREs in the 3′UTR of *Il10* mRNA. **d** HEK-293T cells were co-transfected with pGL3 containing *Il10* promoter and *Rbm47*-expressing or empty vector. After 48 h, the cells were subjected to the dual-luciferase reporter assay. Relative luciferase activity was counted (first fluorescence intensity/second fluorescence intensity). **e** SP2/0 cells were infected with sh-*Rbm47*-expressing or control lentivirus and treated with actinomycin D (10 mg/ml). Total RNA was harvested at 0, 15, 30, 45, or 60 min after treatment. **f** *Il10*-expressing plasmids were co-transfected with *Rbm47*-expressing or empty plasmids into CHO cells, and then the cells were treated with actinomycin D (10 mg/ml). Total RNA was harvested at 0, 15, 30, 45, or 60 min after treatment. *Il10* mRNA levels were measured using q-PCR and normalized to *gadph* mRNA. The normalized level of *Il10* mRNA at time 0 was set to 100. Data are representative of at least three independent experiments, and error bars indicate the standard deviation. **P < 0.01; ***P < 0.001

To address whether Rbm47 can regulate IL-10 by affecting its transcription, we employed a luciferase reporter plasmid containing the *Il10* promoter sequence as previously described. We did not observe any difference in luciferase activity between the *Rbm47*-*GFP*-transfected cells and *GFP*-transfected cells (Supplementary Figure 4), suggesting that Rbm47 has little effect on the transcription of *Il10*. Because Rbm47 contains 3′ RNA recognition motifs, it can recognize specific mRNA molecules and regulate gene expression at the post-transcriptional level. To examine whether Rbm47 can bind to the *Il10* mRNA, we performed RIP experiments. RIP analysis showed that anti-GFP antibody pulled down a greater amount of *Il10* (Fig. 3a) but not *gadph* (Supplementary Figure 5) mRNA than the control IgG, implying that Rbm47 can directly interact with *Il10* mRNA. To investigate the region of the *Il10* mRNA recognized by Rbm47, we co-transfected plasmids containing *Rbm47-GFP* with *Il10*, full-length or *Il10*, 3′UTR and detected precipitated *Il10* mRNA with Rbm47 by RIP experiments. We found that both *Il10*, full-length and *Il10*, 3′UTR mRNA could be precipitated by Rbm47 (Fig. 3b). AREs are classical motifs with a core sequence of AUUUA,^21^ and RNA-binding proteins and microRNAs bind to these elements to regulate the decay of mRNA.^22^ There are nine typical AREs located in the 3′UTR of *Il10* mRNA (Fig. 3c). To test whether Rbm47 interacted with AREs within 3′UTR of *Il10* gene, we co-transfected plasmids containing *Rbm47-GFP* with *Il10*, 3′UTR mRNA in which AREs were eliminated (*Il10*, 3′UTR-dARE). After elimination of AREs, Rbm47 could no longer interact with *Il10*, 3′UTR mRNA (Fig. 3b). Subsequently, we used luciferase reporter systems to further confirm these results. We found that Rbm47 could only downregulate the luciferase activity of the reporter vector with *Il10*, 3′UTR but had no effect on the luciferase activity of reporter vector with *Il10*, 5′UTR, *Il10*, CDS or *Il10*, 3′UTR-dARE (Fig. 3d). Studies have demonstrated that post-transcriptional regulation of mRNA can greatly influence the stability of the IL-10 protein, and Rbm47 may exert a regularly role by stabilizing the *Il10* mRNA.^23, 24^ Thus, we speculated that Rbm47 might affect the stability of *Il10* mRNA in B cells. To validate our hypothesis, actinomycin D was employed to stop transcription. Total RNA was harvested after 0, 15, 30, and 45 min and subjected to q-PCR. The results revealed that knockdown of Rbm47 accelerated the decay of *Il10* mRNA (Fig. 3e); additionally, overexpression of Rbm47 delayed the decay of *Il10* mRNA (Fig. 3f), suggesting that Rbm47 regulates IL-10 gene expression by stabilizing its mRNA in B cells. Taken together, these results demonstrated that Rbm47 positively regulates the stability of *Il10* mRNA by binding to its AREs within the 3′UTR of the *Il10* gene.

### Overexpression of Rbm47 enhanced IL-10 production in B cells

**Fig. 4:**
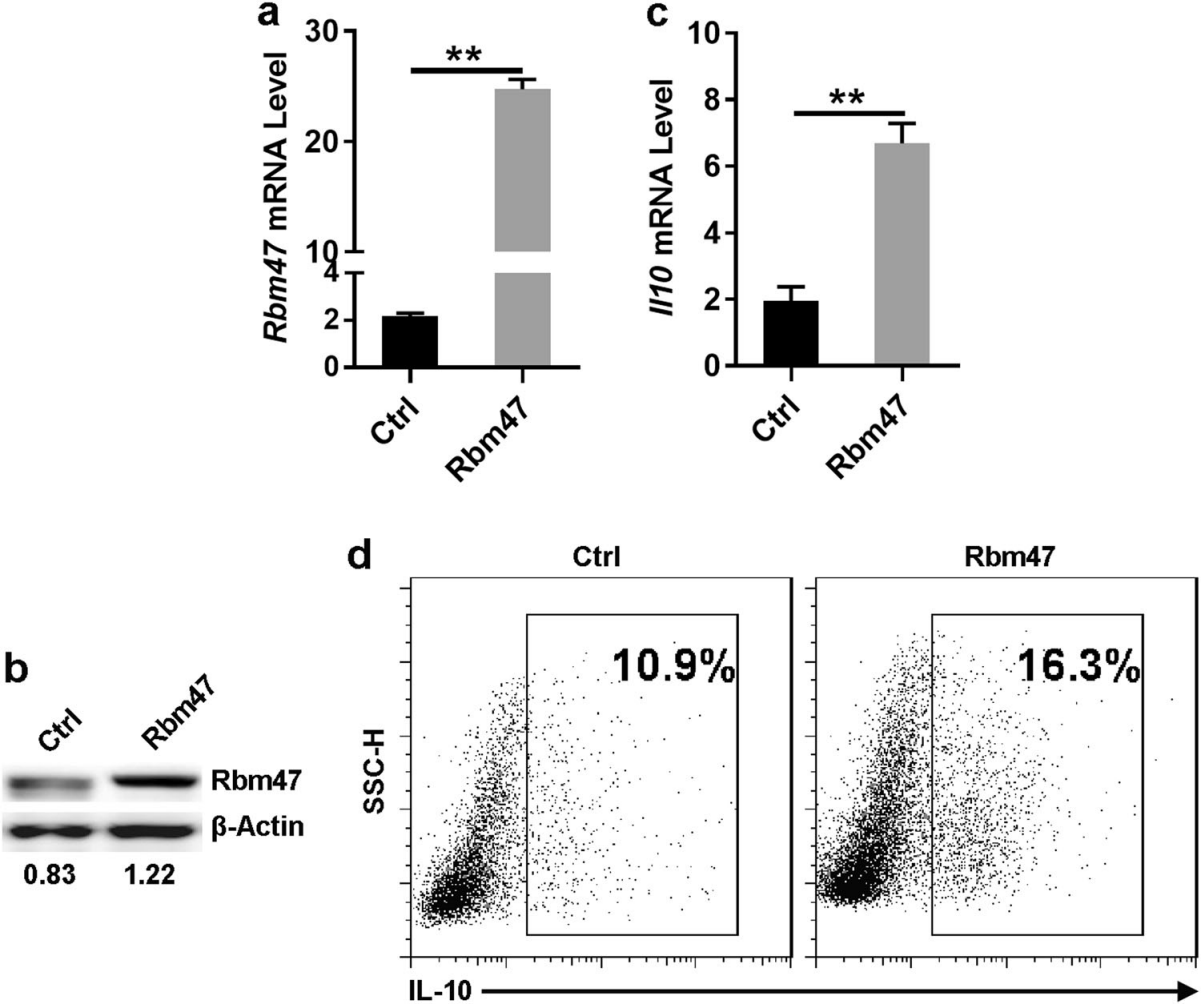
Rbm47 overexpression promotes IL-10 production in B cells. **a**–**d** B cells were isolated from Tiger mice and infected with *Rbm47*-expressing lentiviral particles for 24 h. The infected cells were then washed and cultured for another 3 days in 6-well plates (at 1 × 10^6^ cells/ml, total volume: 5 ml) in the presence of 10 μg/ml LPS. The levels of *Rbm47* (**a**) or *Il10* (**c**) mRNA were detected by q-PCR. The levels of Rbm47 (**b**) or IL-10 (**d**) protein were detected by western blotting or FACS, respectively. Numbers in **b** indicate the ratio of gray values of the corresponding protein to that of β-actin. Data are representative of at least three independent experiments, and error bars indicate the standard deviation. **P < 0.01

To further demonstrate the physiological significance of Rbm47 in the regulation of IL-10 production in B cells, we overexpressed Rbm47 in primary B cells by employing *Rbm47*-expressing lentiviral constructs. The effect of Rbm47 overexpression was confirmed by q-PCR and western blot analysis (Fig. 4a, b). q-PCR analysis showed that Rbm47 overexpression upregulated *Il10* mRNA levels in B cells (Fig. 4c). Furthermore, staining for intracellular cytokines showed that 16.3% of cells infected with *Rbm47*-expressing lentivirus could be induced to express IL-10, which is higher than that in the empty vector-containing lentivirus-infected group (Fig. 4d). Hence, we confirmed that Rbm47 could also positively regulate IL-10 production in primary B cells.

### Overexpression of Rbm47 facilitated immune regulation of B cells

**Fig. 5:**
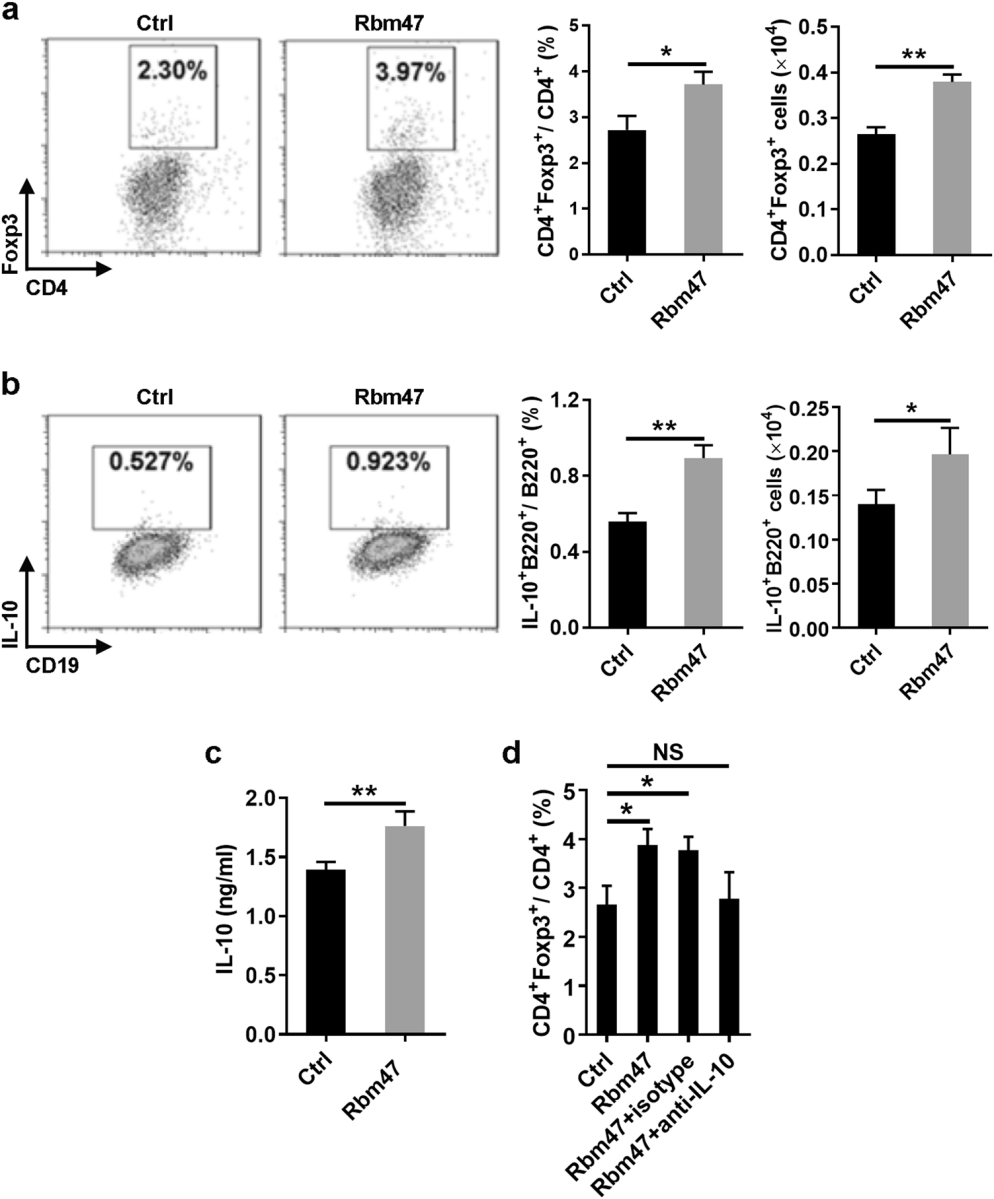
Rbm47-overexpressing B cells facilitate Treg induction in vitro. **a–d** B cells were infected with *Rbm47-GFP*-expressing retroviral particles and sorted by FACS. Sorted cells were co-cultured with CD4^+^ T cells isolated from the spleens of C57BL/6 mice in medium containing anti-CD3, anti-CD28 antibodies and 10 μg/ml LPS. After 72 h, the cells and supernatant were subjected to FACS and ELISA analysis, respectively. The frequency and number of Tregs (**a**), IL-10 positive B cells (**b**), IL-10 concentration in the supernatant (**c**), or frequency of Tregs after neutralization by 10 μg/ml anti-IL-10 antibodies (**d**) are shown. Data are representative of at least three independent experiments, and error bars indicate the standard deviation. *P < 0.05; **P < 0.01; NS not significant

Tregs play a critical role in maintaining immune homeostasis. A number of studies have shown that Bregs exert their function by affecting the role of Tregs.^25^ Numerous studies have shown that IL-10 is very important for the expression of Foxp3 in Tregs.^26^ To evaluate the effect of Rbm47 on the regulatory function of B cells toward CD4^+^Foxp3^+^ T cells, B cells infected with GFP-expressing or *Rbm47*-*GFP*-expressing retroviruses were purified by sorting and then co-cultured with CD4^+^ T cells. Compared with freshly isolated Foxp3^+^CD4^+^ T cells, the percentage of Foxp3^+^CD4^+^ T cells decreased with the duration of culture (data not shown). However, compared with GFP-expressing B cells, co-culture of CD4^+^ T cells with *Rbm47*-*GFP*-expressing B cells resulted in a greater percentage and number of CD4^+^Foxp3^+^ Tregs (Fig. 5a). Again, we found that the percentage and number of IL-10^+^ B cells were higher in *Rbm47*-*GFP*-expressing B cells (Fig. 5b). In addition, we found that the concentration of IL-10 was higher in the culture medium of *Rbm47*-*GFP*-expressing B cells (Fig. 5c). Blockade of IL-10 using anti-IL-10 neutralized antibodies completely abolished the effect of *Rbm47*-*GFP*-expressing B cells on increasing CD4^+^Foxp3^+^ Tregs (Fig. 5d), suggesting that this effect was IL-10 dependent. Moreover, we checked the expression of costimulatory molecules and antigen presentation molecules on the B cells. No differences were found between the *GFP*-expressing B cells and *Rbm47-GFP*-expressing B cells (Supplementary Figure 6), indicating that Rbm47 probably had little effect on the B cell phenotype. Taken together, these results indicated that Rbm47 overexpression might facilitate the regulatory function of B cells in vitro via IL-10.

### Rbm47^+^ B cells suppressed inflammation in mice suffering from colitis

An imbalance in the proportion of Bregs has been observed in intestinal inflammation in mice^27^ and humans,^28^ and transfer of Bregs has been shown to suppress the progression of intestinal inflammation in an IL-10-dependent manner.^6, 27^ We found that the number of IL-10-secreting B cells was reduced in the mesenteric lymph nodes (mLNs) from IL-10-GFP-reporter mice fed with 3% DSS (Supplementary Figure 7a). Consistent with the decreased number of IL-10-expressing B cells, the levels of *Rbm47* and *Il10* mRNA in B cells were also reduced (Supplementary Figure 7b). These findings indicated that the expression of Rbm47 might be related to IL-10 expression in B cells during UC progression.

**Fig. 6:**
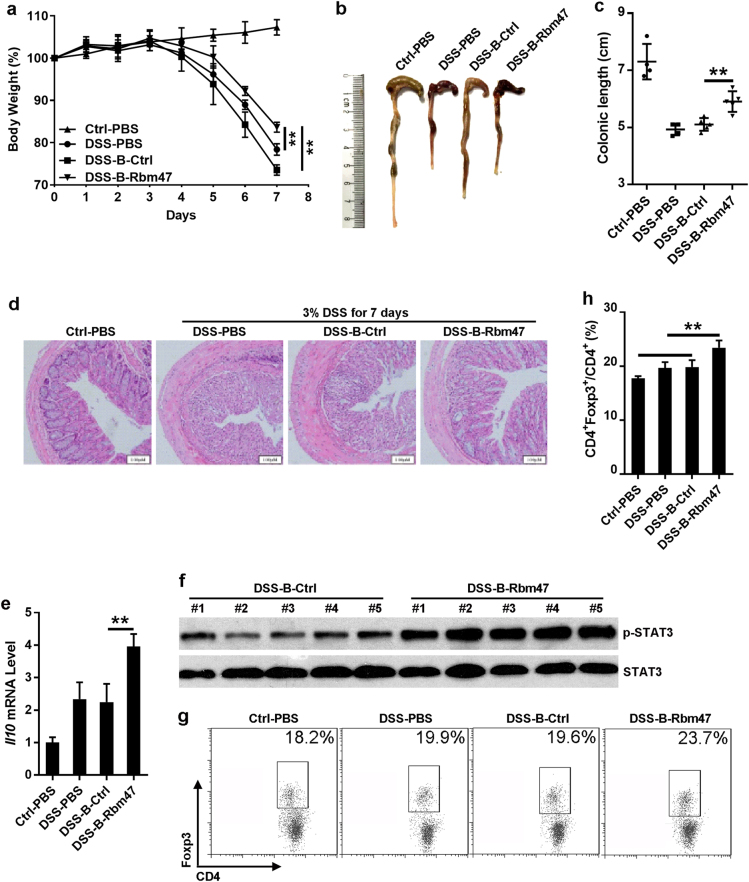
Rbm47-overexpressing B cells reduce the severity of acute colitis. **a–g** For colitis induction, normal drinking water was replaced with sterilized reverse osmotic water containing 3% (w/v) DSS. B cells infected with *GFP* or *Rbm47*-*GFP*-expressing retroviruses were sorted via FACS and intravenously injected into mice (2 × 10^5^ cells/mouse, 5 mice/group) on days 1 and 3 after DSS induction. Body weight was measured daily (**a**). On day 7, the mice were euthanized, and the colonic length was measured (**b**) and statistically analyzed (**c**). The colon samples were subjected to H & E analysis (**d**). *Il10* mRNA levels in colonic tissues were measured by q-PCR (**e**). Phosphorylated STAT3 in colonic tissues was detected by western blotting (**f**). Foxp3^+^CD4^+^ Tregs in mLNs were detected using FACS (**g**) and statistically analyzed (**h**). Scale bar = 100 μm. Data are representative of at least two independent experiments, and error bars indicate the standard deviation. **P < 0.01; NS not significant

To further examine the effect of Rbm47 on the regulatory function of B cells in vivo, C57BL/6 mice were injected with Rbm47-overexpressing B cells on days 1 and 3 after drinking 3% DSS. The body weights of the mice were recorded daily for 7 days, and the mice were subsequently sacrificed to assess the resulting colonic damage. The mice began to lose weight 4 days after the administration of 3% DSS. Notably, the weight loss in mice that received Rbm47-overexpressing B cells was statistically less pronounced than in mice that received control B cells or PBS (Fig. 6a). A reduced colonic length, an indicator of disease severity,^29^ was less severe in mice that received Rbm47-overexpressing B cells (Fig. 6b, c). Histological analysis revealed that the colonic segments from mice exposed to 3% DSS displayed mucosal layer thickening, goblet cell disruption, and leukocyte infiltration. Remarkably, the colonic segments from mice receiving Rbm47-overexpressing B cells displayed histological features comparable with those of healthy mice (Fig. 6d).

To explore the role of IL-10 in this process, we assessed the *Il10* mRNA level in colonic tissues from mice that received control or Rbm47-overexpressing B cells and observed a noticeably enhanced *Il10* mRNA level in colonic tissues from mice that received Rbm47-overexpressing B cells (Fig. 6e). STAT3 is the key downstream transcription factor of IL-10.^30^ We also observed a marked increase in phosphorylated STAT3 in colonic tissues from mice that received Rbm47-overexpressing B cells (Fig. 6f). These results suggested that IL-10 probably participated in the protective effects of Rbm47-overexpressing B cells in UC.

Our above results also showed that Rbm47-overexpressing B cells could facilitate Treg induction in vitro, and numerous studies have shown that Tregs play an important role in various kinds of colitis, including chemically induced intestinal inflammation.^31–33^ We assessed the percentage of Tregs in the mLNs in different groups. The results showed that mice that received Rbm47-overexpressing B cells had a higher proportion of Tregs compared with control mice (Fig. 6g, h), which validated the effect of Rbm47 on the regulatory function of B cells.

We further assessed the expression of α4β7, which is a key gut-specific homing factor for lymphocytes. The results showed no differences in α4β7 expression between mice treated with Rbm47-overexpressing B cells or control B cells (Supplementary Figure 8), suggesting a lack of an effect of Rbm47 on the homing of B cells to the gut. Taken together, these results demonstrated a positive role of Rbm47 in facilitating the regulatory function of B cells in vivo.

## Discussion

Because of the lack of specific markers, Bregs are functionally defined mainly by their ability to secrete IL-10.^34^ Although various mechanisms have been reported to mediate the suppressive functions of Bregs, the IL-10-dependent pathway is still the most important one.^2, 13^ IL-10-defective Bregs fail to exert immune regulatory effects in vitro and in vivo.^6, 15, 16^ In addition, the absence of IL-10-producing Bregs has been implicated in the etiology of numerous immune-related diseases.^35–37^ Thus, revealing the mechanisms of IL-10 regulation in B cells is of great significance. Here we found that Rbm47 positively regulates IL-10 expression at the post-transcriptional level. The results of this study have improved our understanding of the post-transcriptional regulation of IL-10 in Bregs and provide a potential application for Bregs in the treatment of autoimmune diseases.

Rbm47 has been reported to preferentially bind to the 3′UTR of its target mRNAs.^38^ In agreement with these results, we also validated the localization of the Rbm47 binding region in the 3′UTR of *Il10* mRNA. Furthermore, we found that Rbm47 mainly binds to AREs in the 3′UTR of *Il10* mRNA. AREs are classical motifs with a core sequence of AUUUA,^21^ and RNA-binding proteins and microRNAs bind to these elements to regulate the decay of mRNA.^22^ There are 9 typical AREs located in the 3′UTR of *Il10* mRNA, and it has been demonstrated that these motifs are crucial for the stability of *Il10*.^39^ Powell et al. reported that TTP downregulates IL-10 stability by binding to the AREs in its 3′UTR in EL4 and RAW 264.7 cells.^40^ Additionally, macrophages derived from TTP-deficient mice exhibit stronger IL-10 expression.^41^ Moreover, microRNA-4661 binds to the AREs in the 3′UTR of *Il10* mRNA, thus delaying *Il10* mRNA degradation in murine macrophages.^42^ Our results herein reveal that knockdown of Rbm47 accelerates the decay of *Il10* mRNA; moreover, overexpression of Rbm47 delays the decay of *Il10* mRNA. Vanharanta et al. have shown that Rbm47 protects the 3′UTR of mRNAs from destabilizing factors, and Ma et al. have suggested that the stability of *Il10* mRNA can be improved by microRNA-4661 through the inhibition of binding of TTP to ARE motifs.^23, 42^ Thus, we hypothesize that Rbm47 also exerts its protective effects by competing with destabilizers for binding to the AREs on *Il10* mRNA. According to our results, we cannot exclude the possibility that Rbm47 can also bind to other mRNAs and regulate *Il10* mRNA in an indirect manner. The details must be further investigated. However, we believe that this model can explain how IL-10 production in B cells is fine-tuned at the post-transcriptional level.

Tregs play a critical role in maintaining immune homeostasis, and an important function of Bregs is to support the development of Tregs. Different groups have shown that IL-10 is required for Foxp3 maintenance in Tregs.^26, 43^ The transfer of IL-10-sufficient MZ and T2-MZP B cells, but not of IL-10-deficient MZ or follicular B cells, has been shown to restore Treg numbers in B cell-deficient mice.^36, 44^ In our study, Rbm47-overexpressing B cells facilitated Treg induction both in vitro and in vivo along with the upregulation of IL-10.

Ulcerative colitis (UC) is a type of inflammatory bowel disease characterized by chronic gastrointestinal inflammation, which is orchestrated by the production of chemokines and pro-inflammatory cytokines. IL-10 plays a central role in downregulating inflammatory cascades in UC and is likely a candidate for therapeutic intervention. However, the application of IL-10 for UC treatment is hindered by its short half-life and insufficient mucosal penetration.^45^ Increasing evidence has shown that the transfer of IL-10-producing Bregs may be a strategy to alleviate the severity of UC in different models.^6, 27, 46–48^ Oral administration of DSS solution to mice is widely used to mimic human UC because DSS-induced murine colitis shares similar pathological features to the UC observed in patients.^27^ In IL-10-GFP-reporter mice administered 3% DSS, we observed a reduction in the number of IL-10-secreting B cells in mLNs. Moreover, B cells in the mLNs of mice with DSS-induced colitis had a markedly lower expression level of Rbm47 and IL-10. Using the same model, Yanaba et al. have suggested that IL-10-producing B cells expand in the spleen during disease progression.^27^ The discrepancy between their and our findings could be due to the differences in the sites examined. Because the elimination of B cells exacerbates UC in mice and humans,^6, 27, 46, 49, 50^ it is conceivable that Bregs are predominantly enriched relative to effector B cells in inflammatory bowel disease. In fact, our results suggest that the transfer the IL-10^+^ Bregs can effectively suppress inflammation in mice with DSS-induced colitis, indicating that IL-10-producing B cells can reverse the progression of DSS-induced UC. Manipulation of immune cells by various means, such as gene modification of T cells,^51^ dendritic cells,^52^ NK cells^53^ and mesenchymal stem cells,^54^ has been implemented to treat many kinds of diseases. Bregs have attracted the interest of immunologists and clinicians, and investigators have attempted to modify B cells to improve their immune suppressive effect.^55, 56^ Our present results show that overexpression of Rbm47 can promote the immune-suppressive function of B cells in the DSS-induced UC model, which indicates that by manipulating post-transcriptional regulators, the regulatory function of B cell can be improved and the application of Bregs can also be facilitated.

In summary, we successfully identified Rbm47 as a specific post-transcriptional regulator of IL-10 in B cells. Rbm47 likely exerts its regulatory effect by binding to the AREs in the 3′UTR of *Il10* mRNA and protects the transcripts from degradation in B cells. Importantly, Rbm47-overexpressing B cells suppress inflammation in mice suffering from colitis by inducing Tregs. Thus, our study demonstrates that molecules involved in posttranslational regulation may play an important role in Bregs, and gene therapy targeting post-transcriptional regulators such as Rbm47 may be a promising strategy for the application of Bregs in the future.

## Electronic Supplementary material

Supplemental Figures

Supplemental Table
