# Supplementary material for: Post-transcriptional regulator Rbm47 elevates IL-10 production and promotes the immunosuppression of B cells
Source: Cell Mol Immunol. 2018 May 29;16(6):580–9. doi: 10.1038/s41423-018-0041-z (PMC6804925; doi:10.1038/s41423-018-0041-z)
Supplement: Supplementary file 1 — Supplemental Figures [file 41423_2018_41_MOESM1_ESM.docx]

**Supplementary materials**

**
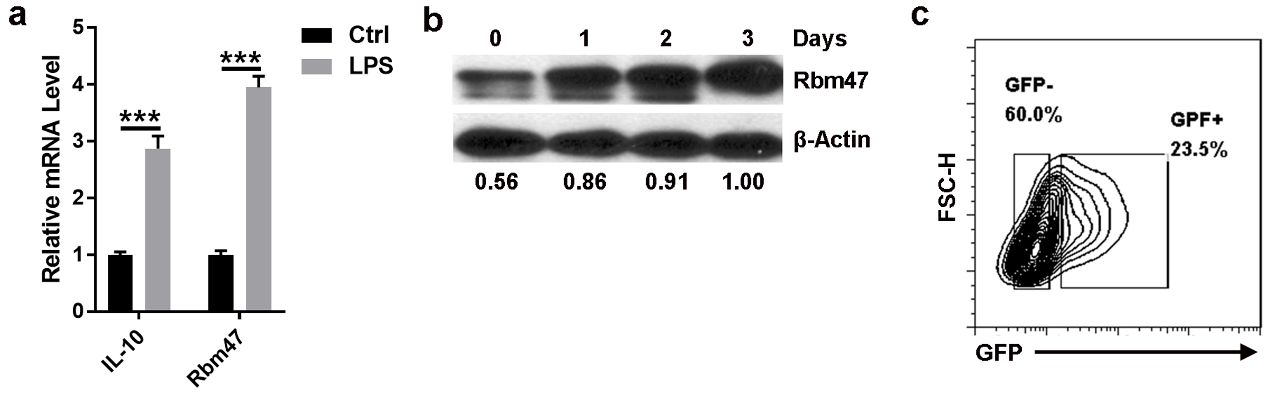
**

**Figure 1** Rbm47 expression in IL-10-producing B cells. (**a, b**) B cells isolated from C57BL/6 mice by MACS were stimulated with 10 µg/ml LPS for 3 days and then *Il10* and *Rbm47* mRNA levels were detected by q-PCR (**a**) or were stimulated with 10 µg/ml LPS for the indicated time and then Rbm47 protein level was detected by western blotting (**b**). (**c**) B cells isolated from the splenocytes of IL-10-GFP reporter mice were stimulated with 10 μg/ml LPS for 3 days followed by FACS to obtain GFP-negative and GFP-positive B cells. Numbers indicate the ratio of gray values of the corresponding protein to that of β-Actin. Data are representative of at least three independent experiments, and error bars indicate standard deviation. ****P* <0.001.


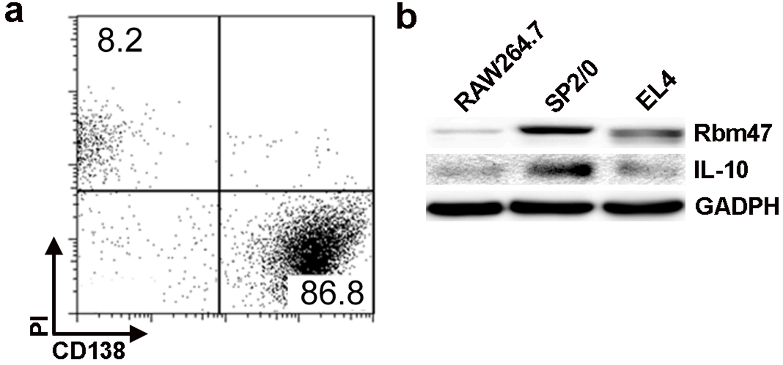


**Figure 2** Characterization of SP2/0 cells. (**a**) After staining with anti-CD138 antibodies and PI, SP2/0 cells were analyzed by FACS. (**b**) Resting RAW 264.7, SP2/0 and EL4 cells were collected and subjected to western blotting analysis for the expression of Rbm47 and IL-10; GADPH was used as the internal reference. Data are representative of at least three independent experiments.


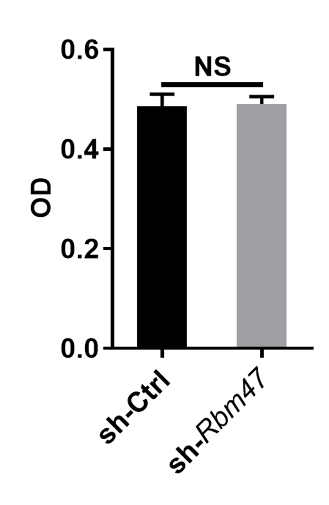


**Figure 3** Proliferation of control and *Rbm47*-specific shRNA-infected SP2/0 cells. Cells from different groups were seeded in equal numbers (1 × 10^5^ cells/ml, total volume: 1 ml) and stimulated with 10 μg/ml LPS. After 3 days, MTT reagent was added followed by OD detection at 450 nm. Data are representative of at least three independent experiments, and error bars indicate standard deviation. NS, not significant.


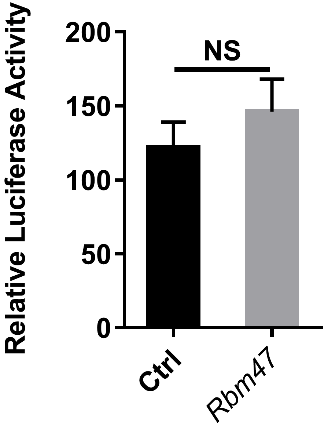


**Figure 4** Rbm47 did not affect transcription of *Il10*. HEK-293T cells were seeded in 6-well plates, 1 × 10^6^ cells per well. After 8~10 hours, pGL3 vectors containing *Il10* promoter sequence were transfected into cells with control or *Rbm47*-expressing pcDNA3.1 vectors. After 48 hours, the cells were subjected to dual-luciferase reporter assay. Relative luciferase activity = (first fluorescence intensity / second fluorescence intensity). Data are representative of at least three independent experiments, and error bars indicate standard deviation. NS, not significant.


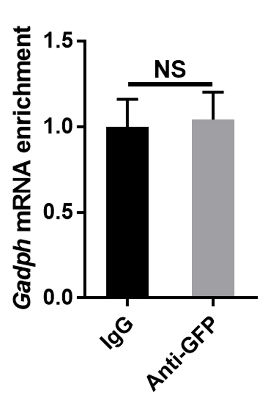


**Figure 5** Rbm47 did not pull down *gadph* mRNA. HEK-293T cells were transfected with GFP- or *Rbm47*-*GFP*-expressing plasmid After 2 days, the cells were harvested and subjected to RIP with IgG and anti-GFP antibody. The precipitated *gadph* mRNA was then reverse transcribed into cDNA and subjected to q-PCR analysis.


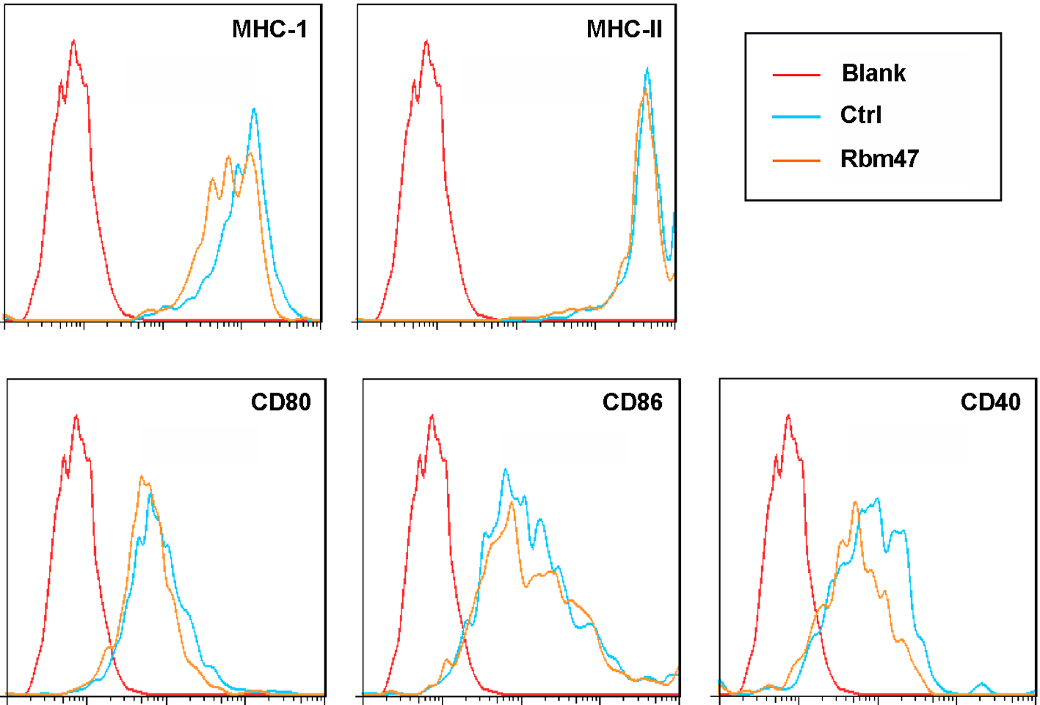


**Figure 6** Rbm47 has negligible influence on the expression of costimulatory and antigen presentation molecules in B cells. B cells infected with *Rbm47-GFP*-expressing or control retroviral particles were subjected to FACS for the indicated markers. Data are representative of at least three independent experiments.


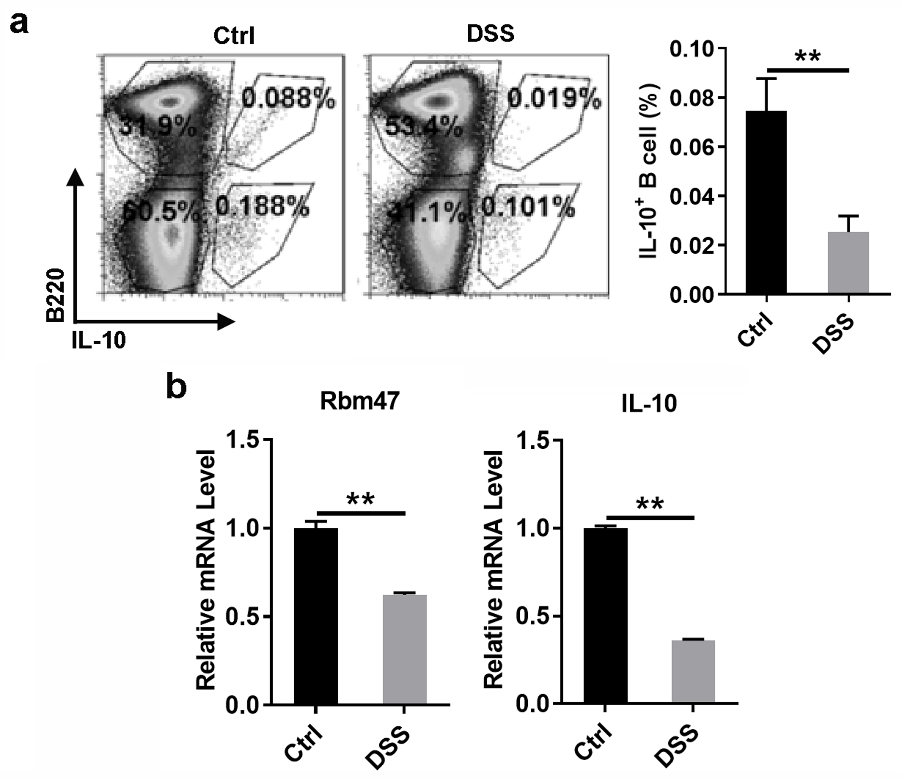


**Figure 7** IL-10-producing B cell numbers are reduced in the mLNs of mice with DSS-induced acute colitis. (**a, b**) IL-10-GFP-reporter mice were given 3% DSS or clean water. Animals were sacrificed 7 days later, and mLNs were subjected to FACS (left) and statistically analyzed (right) (**a**) or B220^+^ cells in mLNs were isolated by microbead followed by q-PCR analysis of *Rbm47* and *Il10* mRNA levels (**b**). Data are representative of at least three independent experiments, and error bars indicate standard deviation. ***P* <0.01.


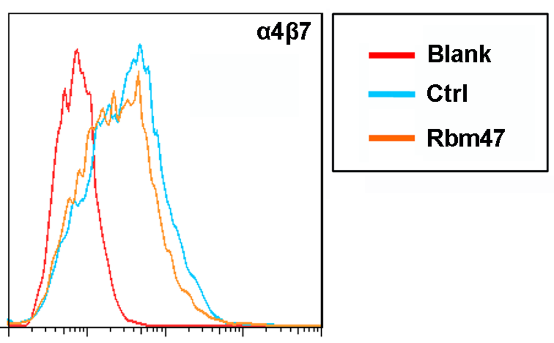


**Figure 8** Rbm47 has negligible influence on the expression of α4β7. B cells infected with *Rbm47*-expressing or control retroviral particles were subjected to FACS analysis of α4β7 expression. Data are representative of at least three independent experiments.
